# Multi-body-site colonization screening cultures for predicting multi-drug resistant Gram-negative and Gram-positive bacteremia in hematological patients

**DOI:** 10.1186/s12879-022-07154-3

**Published:** 2022-02-21

**Authors:** Ignacio Torres, Dixie Huntley, Mar Tormo, Marisa Calabuig, Juan Carlos Hernández-Boluda, María José Terol, Carlos Carretero, Paula de Michelena, Ariadna Pérez, José Luis Piñana, Javier Colomina, Carlos Solano, David Navarro

**Affiliations:** 1 Microbiology Service, Clinic University Hospital, INCLIVA Health Research Institute, Valencia, Spain; 2 Hematology Department, Clinic University Hospital, INCLIVA Health Research Institute, Valencia, Spain; 3 Department of Medicine, School of Medicine, University of Valencia, Valencia, Spain; 4 Department of Microbiology, School of Medicine, University of Valencia, Av. Blasco Ibáñez 17, 46010 Valencia, Spain

**Keywords:** Multi-drug resistant bacteria (MDRB), Colonization, Hematological patients, Bloodstream infection

## Abstract

**Background:**

To investigate the multi-drug resistant bacteria (MDRB) colonization rate in hematological patients hospitalized for any cause using a multi-body-site surveillance approach, and determine the extent to which this screening strategy helped anticipate MDRB bloodstream infections (BSI).

**Methods:**

Single-center retrospective observational study including 361 admissions documented in 250 adult patients. Surveillance cultures of nasal, pharyngeal, axillary and rectal specimens (the latter two combined) were performed at admission and subsequently on a weekly basis. Blood culture samples were incubated in an automated continuous monitoring blood culturing instrument (BACTEC FX).

**Results:**

In total, 3463 surveillance cultures were performed (pharyngeal, n = 1201; axillary-rectal, n = 1200; nasal, n = 1062). MDRB colonization was documented in 122 out of 361 (33.7%) admissions corresponding to 86 patients (34.4%). A total of 149 MDRB were isolated from one or more body sites, of which most were Gram-negative bacteria, most frequently non-fermenting (n = 83) followed by *Enterobacterales* (n = 51). BSI were documented in 102 admissions (28%) involving 87 patients. Overall, the rate of BSI caused by MDRB was significantly higher (*p* = 0.04) in the presence of colonizing MDRB (16 out of 47 admissions in 14 patients) than in its absence (9 out of 55 admissions in 9 patients). Colonization by any MDRB was independently associated with increased risk of MDRB-BSI (HR, 3.70; 95% CI, 1.38–9.90; *p* = 0.009).

**Conclusion:**

MDRB colonization is a frequent event in hematological patients hospitalized for any reason and is associated with an increased risk of MDRB BSI. The data lend support to the use of MDRB colonization surveillance cultures for predicting the occurrence of MDRB BSI in this cohort.

## Background

Bloodstream infections (BSI) caused by multidrug-resistant bacteria (MDRB), particularly those involving extended-spectrum beta-lactamase (ESBL)- or carbapenemase-producing *Enterobacterales,* MDR *Pseudomonas aeruginosa*, and vancomycin-resistant enterococci (VRE), pose a major threat for patients with hematological malignancies undergoing chemotherapy or transplantation due to their inherent associated morbidity and mortality [1–8]. Hematological patients are at increased risk of MRDB BSI as a result of extensive broad-spectrum antibiotics use, prolonged hospitalizations, and disruption of mucosal surfaces and neutropenia due to cytotoxic chemotherapy [1–3]. Identifying MDRB-colonized patients through active surveillance could facilitate appropriate or early adjustment of empirical therapy for BSI, yet data supporting this assumption have been inconsistent [9–17].

Nose and rectum are the preferred sites for surveillance of methicillin-resistant *Staphylococcus aureus* (MRSA) and VRE/MDR Gram-negative bacteria, respectively [18–23]; nonetheless, there is no consensus regarding anatomical sites to be sampled for MDR Gram-negative bacteria screening, microbiological methods for the purpose, or the benefit of performing screening cultures targeting certain MDR bacteria (i.e. MDR-*Stenotrophomonas maltophilia*) [18, 24]. This was highlighted in a recent study revealing striking differences across Spanish centers in sampling protocols, body sites chosen for screening and microbiological testing procedures [25]. The purpose of the current study was twofold. First, we investigated the MDRB colonization rate in a series of hematological patients hospitalized for any cause using a multi-body-site surveillance approach and identified risk factors for this event. Second, we ascertained the incidence of MDRB BSI in patients with or without previous detection of MDRB colonization and determined whether the above screening strategy permitted anticipation of its occurrence.

## Methods

### Study population

In this single-center retrospective observational study we included a total of 361 admissions to the hematology wards from January 2015 to December 2019, involving 250 adult (> 18 years) hematological patients. No exclusion criteria were established. Twenty-nine patients were eventually admitted to the Intensive Care Unit (ICU). The cohort comprised patients with the following underlying diseases: lymphoma (n = 93), acute myeloid leukemia (n = 69), multiple myeloma (n = 45), acute lymphoblastic leukemia (n = 16), myelodysplastic syndrome (n = 11), chronic lymphocytic leukemia (n = 2), chronic myeloid leukemia (n = 1) and others (n = 13). Reasons for hospital admission were receipt of allogeneic or autologous stem cell transplantation (n = 163), treatment of underlying disease (n = 136), neutropenic fever (n = 35), performance of diagnostic procedures (n = 9) acute or chronic graft versus host disease (n = 7), and other causes (n = 11). For patients with two or more admissions (n = 59), these took place at least 3 months apart. Colonization by the same one or more MDRB in two or more consecutive admissions for a given patient was considered as a single episode. Median hospital stay was 25 days (range, 4–96). Baseline characteristics, clinical data, recent antibiotic treatment and clinical outcomes were registered.

### Microbiological analyses

During patient hospitalization, surveillance cultures of nasal, pharyngeal, axillary and rectal specimens were scheduled to be performed systematically within 48 h of admission (baseline) and on a weekly basis afterwards, as a part of routine patient care and according to predefined protocols, as detailed below. All patients colonized with MDRB underwent contact isolation. Surveillance specimens were delivered in AMIES transport medium (cliniswab^LTS^, Aptaca Spa., Canelli, Italy) to the Microbiology Service and immediately processed in accordance with the Procedures in Clinical Microbiology guidelines issued by the Spanish Society of Infectious Diseases and Clinical Microbiology (SEIMC) [26]. Swabs were placed in brain–heart infusion broth tubes (BHI; Oxoid Limited, Hampshire, UK) containing a disc of either cefotaxime (30 µg) or imipenem (10 µg), for MDR-Gram negative enrichment and incubated at 37 °C in a CO_2_ incubator (Heracell 240i CO_2_ incubator, Thermo Fisher Scientific, Langenselbold, Germany) for 24 h. Pharyngeal and nasal swabs were processed individually, while axillary and rectal swabs were combined, as previous data from our group indicated that this strategy yielded comparable results to those obtained by processing both of these specimens individually (not shown) and resulted in lower cost burden. Specimens were subcultured on MacConkey Agar (Becton Dickinson, New Jersey, USA) in which imipenem (10 µg), cefotaxime (30 µg) and ceftazidime (30 µg) discs (Oxoid Limited, Hampshire, UK) were placed and Columbia Blood Agar with 5% Sheep Blood (Becton Dickinson) in which oxacillin (1 μg) and 30 μg vancomycin discs (Oxoid Limited) were placed. Isolated colonies growing near the discs were identified using Matrix-Assisted Laser Desorption/Ionization time-of-flight Mass spectroscopy -MALDI-TOF MS- (Bruker Daltonics, MA, USA). Conventional antimicrobial susceptibility testing from isolated bacteria was performed by broth microdilution using the MicroScan NM44 panel for Gram-negative bacteria and PM33 for Gram-positive bacteria, both from Beckman Coulter (Brea, CA, USA), and interpreted according to contemporary European Committee on Antimicrobial Susceptibility Testing (EUCAST) guidelines https://www.eucast.org/fileadmin/src/media/PDFs/EUCAST_files/Breakpoint_tables/v_10.0_breakpoint_Tables.pdf.). The Antimicrobial Resistance (AMR) Direct Flow Chip (Máster Diagnóstica, Granada, Spain), DNA microarray-based assay was used for antimicrobial resistance gene characterization from bacterial isolated colonies, as described [28] and was performed either contemporarily or retrospectively.

Blood culture samples (BACTEC Plus Aerobic/F and Plus Anaerobic/F medium bottles, Becton Dickinson—BD—and Company, New Jersey, USA) were collected from patients with suspected bacteremia and incubated in an automated continuous monitoring blood culturing instrument (BACTEC FX; BD). Aliquots from each positive BC bottle were subjected to routine Gram stain microscopy, subcultured on chocolate blood medium (BD) and incubated for 24–48 h. From 2018 onwards, direct bacterial identification from BCs was performed by MALDI-TOF MS testing using intact bacteria, as previously described [27].

### Definitions

MDRB was defined as resistance to one or more agents in three or more antimicrobial categories, as previously recommended [29]. The MDRB targeted in screening cultures were ESBL-carbapenemase- and plasmidic *AmpC*-producing *Enterobacterales*, MDR-*P. aeruginosa*, MDR-*S. maltophilia*, MDR-*Acinetobacter* spp. MRSA and VRE. A febrile neutropenia episode was defined as a single oral temperature of ≥ 38.3 °C or a temperature of ≥ 38.0 °C (100.4°F) sustained over 1 hour with an absolute neutrophil count < 500 cells/mm^3^ [1]. Administration of any systemic antibiotic within one month prior to admission was considered prior antibiotic therapy [1, 12]. MDRB colonization was defined as the detection of the respective organism in at least one surveillance culture (from any site). Empirical antibiotic therapy (antibiotic administration without prior identification of the causative bacteria) was initiated at physician discretion according to local guidelines, which take into consideration several factors including baseline risk for severe infection (i.e. neutropenia), site of infection and previous records of MDRB colonization. MDR Gram-negative bacteria were covered by administering beta-lactam antibiotics with antipseudomonal activity (usually, piperacillin-tazobactam or carbapenems) either in monotherapy or in combination with aminoglycosides. When appropriate, MDR Gram positive bacteria were covered by adding to the aforementioned regimens either vancomycin, linezolid or daptomycin. Similarity between susceptibility antimicrobial profile and genotypic resistant marker pattern among isolates recovered from blood and surveillance cultures was deemed to indicate bacterial identity.

### Statistical analysis

Frequency comparisons for categorical variables were carried out using the Fisher exact test or the Chi-square test when appropriate. A *P*-value < 0.05 was considered statistically significant. Odds ratios (OR) and hazard ratios (HR) were determined by Cox and logistic regression analyses, respectively. For multivariate analyses, only variables with parameter estimates showing a *P* value ≤ 0.10 in univariate analyses were included; two-sided *P*-values < 0.05 were deemed to be significant. Analyses were performed using SPSS version 25.0 (SPSS, Chicago, IL, USA).

## Results

### Colonization by MRDB in hematological patients

A total of 3463 surveillance cultures (pharyngeal, n = 1201; axillary-rectal, n = 1200; nasal, n = 1062) were performed during the study period in hematological patients included in the study. MDRB colonization was documented in 122 out of 361 (33.7%) admissions corresponding to 86 out of 250 patients (34.4%), of whom 75 were hospitalized at the hematology ward (111 out of 332 admissions; 33.4%) and 11 at ICU (11 out of 29 admissions; 37.9%).

A total of 149 MDRB were isolated from one or more body sites (Table 1), of which most were Gram-negative bacteria, most frequently non-fermenting (n = 83) followed by *Enterobacterales* (n = 51). MDR Gram-positive bacteria (MRSA or VRE-*Enterococcus faecium* with vancomycin and teicoplanin MICs > 16 mg/L) were isolated in 15 admissions. In most cases, MDRB were cultured from a single body site (82/122; 67.2%), irrespective of the hospitalization ward (68.5% in hematology and 54.5% in ICU).

**Table 1:** Multi-drug resistant bacteria (MDRB) isolated from surveillance colonization cultures

Any MDRB	149 (100)
Gram-negative bacteria	134 (89.9)
*Enterobacterales*	51 (34.2)
ESBL-*Escherichia coli*	36 (24.1)
Plasmidic *AmpC Escherichia coli*	5 (3.4)
ESBL-*Klebsiella pneumoniae*	6 (4)
Plasmidic *AmpC Klebsiella pneumoniae*	2 (1.3)
ESBL-*Enterobacter cloacae*	1 (0.7)
Class B carbapenemase (VIM type) *Klebsiella pneumoniae*	1 (0.7)
Non-fermenting Gram-negative bacteria	83 (55.7)
MDR-*Pseudomonas aeruginosa*	37 (24.8)
Class B carbapenemase (VIM type) *Pseudomonas aeruginosa*	16 (10.8)
MDR-*Acinetobacter* spp.	3 (2)
MDR-*Stenotrophomonas maltophilia*	27 (18.1)
Gram-positive bacteria	15 (10.1)
MRSA	6 (4)
VRE	9 (6.1)

*ESBL* extended spectrum β-lactamase, *MDR* multidrug-resistant, *MRSA* methicillin-resistant *Staphylococcus aureus*, *VRE* vancomycin-resistant enterococci

When considering all hospital admissions, one or more colonizing MDRB were present in 47 out of 329 available baseline specimens (14.2%) corresponding to 36 patients: ESBL-producing *Enterobacterales* (n = 22)*,* MDR-*P. aeruginosa* (n = 10), MDR-*Stenotrophomonas maltophilia* (n = 8), MRSA (n = 4) and others (n = 8).

Interestingly, among patients with more than one hospital admission (n = 59), 14 tested negative during the first hospital stay and became colonized during the second one. All these patients received broad-spectrum antimicrobial therapy during the first hospital admission period. Nevertheless, the median length of hospital stay in first admissions was not significantly different (*P* = 0.27) between those who became colonized in subsequent admission periods (31 days; range, 6–64) and those who did not (27 days; range, 18–92).

Overall, recovery of any MDRB was consistently more likely from axillary-rectal specimens than from pharyngeal or nasal specimens, at both the hematology ward and ICU, as shown in Table 2. ESBL-producing *Enterobacterales* (specially *E. coli*) and MDR-*P. aeruginosa* represented the commonest MDRB recovered from both axillary-rectal specimens and pharyngeal specimens. MDR-*S. maltophilia* was cultured more frequently from pharyngeal than from axillary-rectal specimens. VRE were recovered at the same rate from axillary-rectal and pharyngeal specimens.

**Table 2:** Multi-drug resistant bacteria (MDRB) isolated from surveillance colonization cultures

MDRB	Specimen from which MDRB were isolated in patients admitted to the hematology ward/Intensive care unit		
	Pharyngeal, ward, no. (%) / ICU, no. (%)	Nasal, ward, no. (%) / ICU, no. (%)	Axillary-rectal, ward, no. (%) / ICU, no. (%)
Any MDRB	59 (17.7) / 6 (20.6)	13 (3.9) / 4 (13.7)	97 (29.2) / 9 (31.0)
Gram-negative bacteria	54 (16.2) / 6 (20.6)	7 (2.1) / 4 (13.7)	89 (26.8) / 9 (31.0)
*Enterobacterales*	11 (3.3) / 1 (3.4)	0 (0) / 2 (6.8)	42 (12.6) / 6 (20.6)
ESBL-*Escherichia coli*	7 (2.1) / 1 (3.4)	0 (0) / 1 (3.4)	30 (9.0) / 5 (26.2)
Plasmidic *AmpC Escherichia coli*	0 (0) / 0 (0)	0 (0)	4 (1.2) / 1 (11.1)
ESBL-*Klebsiella pneumoniae*	3 (0.9) / 0 (0)	0 (0)	6 (1.8) / 0 (0)
Plasmidic *AmpC Klebsiella pneumoniae*	0 (0) / 0 (0)	0 (0)	2 (0.6) / 0 (0)
ESBL-*Enterobacter cloacae*	1 (0.3) / 0 (0)	0 (0)	0 (0) / 0 (0)
Class B carbapenemase (VIM type) *Klebsiella pneumoniae*	0 (0) / 0 (0)	0 (0) / 1 (3.4)	0 (0) / 0 (0)
Non-fermenting Gram-negative bacteria	43 (12.9) / 5 (17.2)	7 (2.1) / 2 (6.8)	47 (14.1) / 3 (10.3)
MDR-*Pseudomonas aeruginosa*	17 (5.1) / 3 (10.3)	3 (0.9) / 0 (0)	24 (7.1) / 2 (6.8)
Class B carbapenemase (VIM type) *Pseudomonas aeruginosa*	6 (1.8) / 0 (0)	0 (0) / 0 (0)	15 (4.5) / 0 (0)
MDR-*Acinetobacter* spp	1 (0.3) / 0 (0)	1 (0.3) / 0 (0)	1 (0.3) / 0 (0)
MDR-*Stenotrophomonas maltophilia*	19 (5.7) / 2 (6.8)	3 (0.9) / 2 (6.8)	7 (2.1) / 1 (3.4)
Gram-positive bacteria	5 (1.5) / 0 (0)	6 (1.8) / 0 (0)	8 (2.4) / 0 (0)
MRSA	0 (0) / 0 (0)	6 (1.8) / 0 (0)	0 (0) / 0 (0)
VRE	5 (1.5) / 0 (0)	0 (0) / 0 (0)	8 (2.4) / 0 (0)

*ESBL* extended spectrum β-lactamase, *MDR* multidrug-resistant, *MRSA* methicillin-resistant *Staphylococcus aureus*, *VRE* vancomycin-resistant enterococci

Surveillance of pharyngeal and nasal sites enabled documentation of colonization by one or more MDRB (n = 41) that were absent from axillary-rectal cultures in 31 out of the 122 (25.4%) admissions (Table 3). In most cases, these were MDR-non-fermenting Gram-negative bacteria, in particular MDR-*P. aeruginosa* and MDR-*S. maltophilia* recovered from nasal and/or pharyngeal specimens. In contrast, screening of pharyngeal and nasal sites seldom increased the detection rate of MDR-*Enterobacterale*s or VRE colonization provided by axillary-rectal specimens. As expected, nasal site screening allowed recovery of MRSA which could not be cultured from the other specimen types.

**Table 3:** Multi-drug resistant bacteria (MDRB) isolated from pharyngeal, nasal specimens or both and missed by axillary-rectal surveillance cultures

MDRB	Specimen from which MDRB were isolated	
	Pharyngeal, no	Nasal, no
Any MDRB	33	13
Gram-negative bacteria	32	7
*Enterobacterales*		
ESBL-*Escherichia coli*	2	0
ESBL-*Enterobacter cloacae*	1	0
Class B carbapenemase (VIM type) *Klebsiella pneumoniae*	0	0
Non-fermenting		
MDR-*Pseudomonas aeruginosa*	11	1
Class B carbapenemase (VIM type) *Pseudomonas aeruginosa*	1	0
MDR-*Acinetobacter* spp.	1	1
MDR-*Stenotrophomonas maltophilia*	16	4
Gram-positive bacteria	1	6
MRSA	0	6
VRE	1	0

*ESBL* extended spectrum β-lactamase, *MDR* multidrug-resistant, *MRSA* methicillin-resistant *Staphylococcus aureus*, *VRE* vancomycin-resistant enterococci

### BSI in patients colonized with MDRB

**Fig. 1:**
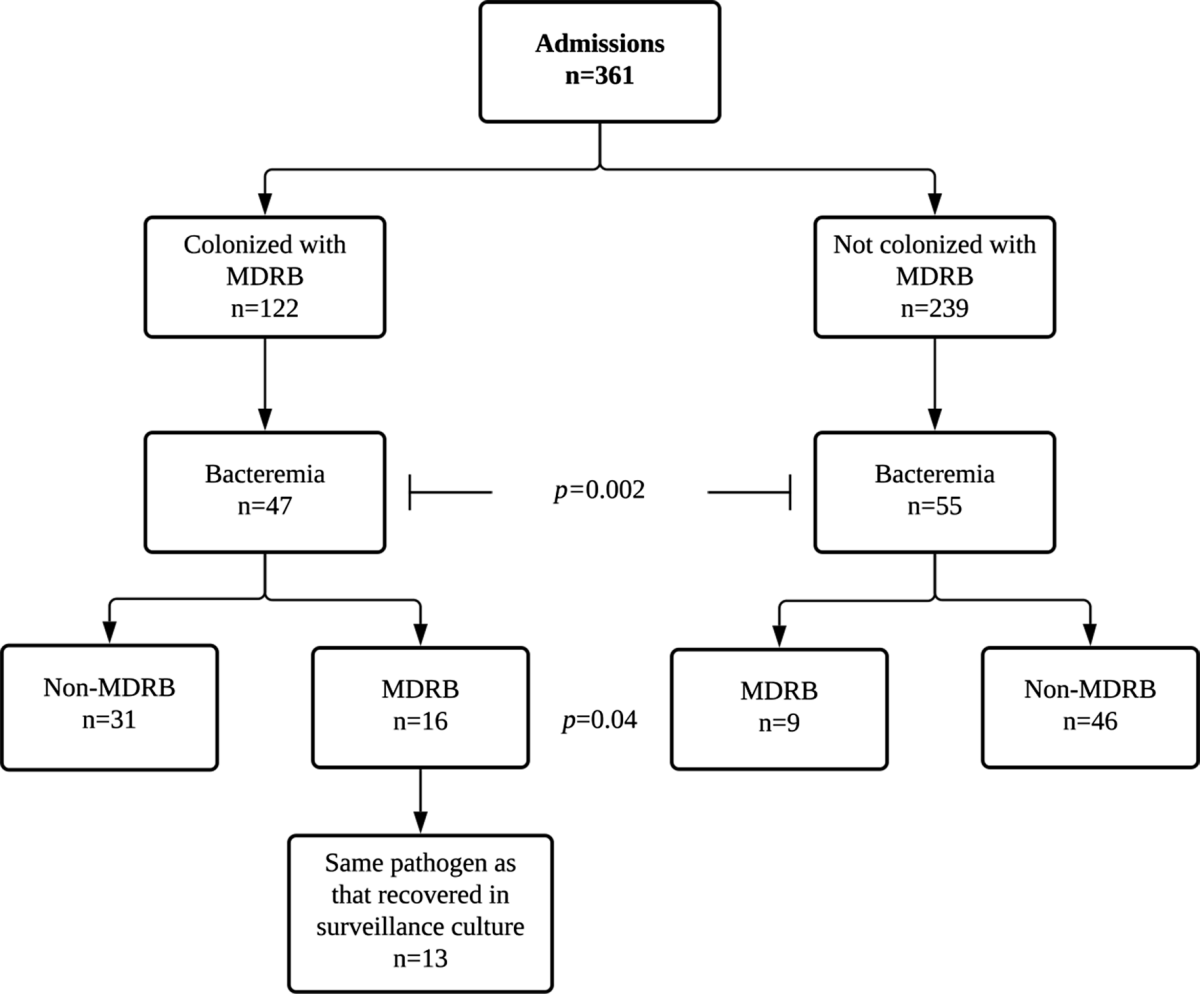
Flow chart depicting relevant data on bloodstream infections occurring in hematological patients either colonized or not with multi-drug resistant bacteria (MDRB)

As depicted in Fig. 1, BSI were documented in 102 admissions (28%) from 87 patients, of which 98 were monomicrobial and 4 polymicrobial. Therefore, a total of 107 isolates were recovered from BC (Table 4). There were 47 BSI episodes (39 patients) among 122 hospitalizations in which colonizing MDRB were identified in the last surveillance cultures processed prior to BSI detection (within 2–7 days), and 55 (52 patients) among 239 admissions in which colonizing MDRB were not documented (*p* = 0.002). Overall, the rate of BSI caused by MDRB was significantly higher in the presence of colonizing MDRB (16 out of 47, in 14 patients) than in its absence (9 out of 55, in 9 patients) (*p* = 0.04). Out of the 16 BSI occurring in MDRB-colonized patients, 13 (in 11 patients) were deemed to be due to the colonizing isolate (ESBL-producing *E. coli*, n = 5; plasmidic *AmpC*-producing *E. coli,* n = 2*;* MDR *P. aeruginosa,* n = 2*;* class B carbapenemase (VIM type)-producing *P. aeruginosa*, n = 2; ESBL-producing *K. pneumoniae,* n = 1*;* VRE, n = 1). Colonization by any MDRB was associated with an increased risk of MDRB BSI (HR, 3.70; 95% CI, 1.38–9.90; *p* = 0.009) in multivariate models adjusted for age, sex, underlying hematological disease, receipt of transplant and previous antibiotic treatments. Colonization by MDRB had a positive and negative predictive value of 68.5% and 64% for the occurrence of MDRB-BSI, respectively. The 13 colonizing MDRB causing BSI could be recovered from one or more body sites (Table 5). Interestingly, all but one of these isolates (MDR-*P. aeruginosa*) were cultured from axillary-rectal screening cultures.

**Table 4:** Bacteria isolated from blood cultures

All isolates (%)	107
Gram-negative bacteria	46 (43)
*Enterobacterales*	36 (33.7)
*Citrobacter freundii*	1 (0.9)
*Escherichia coli*	20 (18.8)
*Klebsiella oxytoca*	1 (0.9)
*Serratia marcescens*	1 (0.9)
ESBL-*E. coli*	9 (8.5)
ESBL-*K. pneumoniae*	1 (0.9)
Plasmidic *AmpC E. coli*	3 (2.8)
Non-fermenting	10 (9.3)
Non MDR-*Pseudomonas aeruginosa*	3 (2.8)
MDR-*Pseudomonas aeruginosa*	3 (2.8)
*C*lass B carbapenemase (VIM type) *Pseudomonas aeruginosa*	3 (2.8)
MDR-*Stenotrophomonas maltophilia*	1 (0.9)
Gram-positive bacteria	58 (54.2)
CNS	22 (20.5)
MR-CNS	10 (9.3)
MRSA	4 (3.7)
*Enterococcus* spp.	19 (17.9)
VRE	1 (0.9)
*Streptococcus* spp.	2 (1.9)
Other microorganisms	3 (2.8)
*Candida* spp.	2 (1.9)
*C. perfringens*	1 (0.9)

*ESBL* extended spectrum β-lactamase, *MDR* multidrug-resistant, *CNS* coagulase-negative *Staphylococcus* spp, *MR-CNS* methicillin-resistant coagulase-negative *Staphylococcus* spp, *MRSA* methicillin-resistant *Staphylococcus aureus*, *VRE* vancomycin-resistant enterococci

**Table 5:** Multi-drug resistant bacteria (MDRB) isolated from surveillance colonization cultures causing bloodstream infection

MDRB	Specimen from which MDRB were isolated		
	Pharyngeal, no. (%)	Nasal, no. (%)	Axillary-rectal, no. (%)
Any MDRB	5 (100)	1 (100)	12 (100)
Gram-negative bacteria	5 (100)	1 (100)	11 (91.7)
*Enterobacterales*	2 (40)	1 (100)	8 (66.7)
ESBL-*Escherichia coli*	1 (20)	1 (100)	5 (41.7)
Plasmidic *AmpC Escherichia*	0 (0)	0 (0)	2 (16.7)
ESBL-*Klebsiella pneumoniae*	1 (20)	0 (0)	1 (8.3)
Non-fermenting Gram-negative bacteria	3 (60)	0 (0)	3 (25)
MDR-*Pseudomonas aeruginosa*	1 (20)	0 (0)	1 (8.3)
Class B carbapenemase (VIM type) *Pseudomonas aeruginosa*	2 (40)	0 (0)	2 (16.7)
Gram-positive bacteria	0 (0)	0 (0)	1 (8.3)
VRE	0 (0)	0 (0)	1 (8.3)

*ESBL* extended spectrum β-lactamase, *MDR* multidrug-resistant, *VRE* vancomycin-resistant enterococci

## Risk factors for MDRB colonization in hematological patients

We next investigated whether MDRB colonization was associated with any demographic or clinical characteristic of patients during hospital admission (Table 6). Frequency comparison analyses revealed statistically significant differences between colonized and non-colonized cases when comparing sex (*p* = 0.005), underlying hematological malignancy (*p* = 0.002), with increased MDRB colonization rate among patients with acute myeloid leukemia, and whether the patient had undergone hematopoietic stem cell transplantation (*p* = 0.025). A trend towards a higher MDRB colonization rate was seen in older patients (*p* = 0.06) and those who had been treated with antibiotics within the month prior to admission (*p* = 0.07). None of these factors except for sex and acute myeloid leukemia as the underlying disease were independently associated with MDRB colonization (Table 7).

**Table 6:** Risk factors for multi-drug bacteria (MDRB) colonization during hospital admissions

Demographic parameters	No MDRB colonization no. (%)	MDRB colonization n (%)	*P* value
Age^a^			
≤ 58	140 (58.6)	59 (48.4)	0.06
> 58	99 (41.4)	63 (51.6)	
Sex			
Male	130 (54.4)	85 (69.7)	0.005
Female	109 (45.6)	37 (30.3)	
Diagnosis			0.002
AA	1 (0.4)	0 (0)	
Lymphoma	82 (34.3)	20 (16.4)	
ALL	21 (8.8)	8 (6.6)	
CLL/LPD	2 (0.8)	0 (0)	
AML	81 (33.9)	68 (55.7)	
CML	0	1 (0.8)	
MM	33 (13.8)	13 (10.7)	
MDS	9 (3.8)	5 (4.1)	
Other	10 (4.2)	7 (5.7)	
Urinary catheter			0.64
No	218 (91.2)	113 (92.6)	
Yes	21 (8.8)	9 (7.4)	
Chemotherapy			0.15
No	40 (16.7)	28 (23)	
Yes	199 (83.3)	94 (77)	
Peripheral or central catheter			0.98
No	2 (0.8)	1 (0.8)	
Yes	237 (99.2)	121 (99.2)	
Previous antibiotic treatment			0.07
No	164 (68.6)	72 (59)	
Yes	75 (31.4)	50 (41)	
Allogeneic or autologous hematopoietic stem cell transplantation			0.02
No	90 (37.7)	61 (50)	
Yes	149 (62.3)	61 (50)	

*AA* aplastic anemia, *ALL* acute lymphoblastic leukemia, *CLL/LPD* chronic lymphocytic leukemia/lymphoproliferative disorders, *AML* acute myeloid leukemia, *CML* chronic myeloid leukemia, *MM* multiple myeloma, *MDS* myelodysplastic syndrome

^a^Median age of patients at admission

**Table 7:** Risk factors for multi-drug resistant bacteria (MDRB) colonization during hospital admission: logistic regression analysis

Parameter	Univariate			Multivariate		
	OR	CI 95%	*P*-value	OR	CI 95%	*P*-value
Age (> 58 vs. ≤ 58)	1.51	0.97–2.341	0.06	1.45	0.91–2.29	0.11
Sex (male vs. female)	1.92	1.21–3.05	0.005	1.69	1.04–2.74	0.03
Acute myeloid leukemia (yes vs. no)	2.45	1.57–3.83	< 0.001	2.3	1.32–4.00	0.03
Previous antibiotic treatment (yes vs. no)	1.51	0.96–2.38	0.07	1.35	0.84–2.19	0.21
Transplant (yes vs. no)	1.65	1.06–2.57	0.02	0.92	0.52–1.63	0.79

*OR* odds ratio

## Discussion

Several factors mainly including broad-spectrum antibiotics use and prolonged hospital stays pose hematological patients at increased risk of MRDB colonization [1–3]. In turn, disruption of mucosal surfaces and neutropenia due to cytotoxic chemotherapy or transplantation favor the occurrence of MDRB BSI, which are associated with high morbidity and mortality [1–8]. In this study, we used a multi-body site screening strategy to investigate the rate of MDRB colonization in hematological patients admitted to the hospital for any cause (most frequently receipt of allogeneic or autologous stem cell transplantation, treatment of underlying disease or neutropenic fever) and to what extent this approach allowed us to predict MDRB BSI. Detection of genotypic determinants of antimicrobial resistance in MDRB was performed using a DNA-based microarray, which displays results that fully agree with genome sequencing data [26]. In line with previous studies [30, 31], to increase the rate of detection of MDRB we performed a combination of upper respiratory tract, gastrointestinal tract and axillar sampling. We documented colonization by one or more MDRB, mostly ESBL-producing *Enterobacterales* and MDR *P. aeruginosa* (including VIM-type carbapenemase producers) and *S. maltophilia*, in 33.7% of admissions, corresponding to 34.4% of patients in this series. Although MDRB colonization was more frequently documented during hospital stay, at either hematology ward or ICU, a non-negligible percentage of patients (14% of admissions) were identified as being colonized at baseline. Of note, colonization by carbapenemase-producing *Enterobacterales* and VRE was anecdotal, as expected considering the very low prevalence of these MDRB in our setting (not shown). Direct comparison of the current study with others addressing this same issue [9–17] is simply unfeasible for a number of reasons, including differences across studies in patients characteristics, local epidemiology particularities and notably targeted MDRBs, screening strategy and methodology used for MDRB detection, all of which may impact dramatically on results. Regarding the latter, for example, we sampled the upper respiratory tract (pharyngeal and nasal specimens), axillary and rectal sites and combined rectal and axillary specimens, while others used only rectal specimens for surveillance. Although MDRB recovery was more likely from axillary-rectal specimens than from upper respiratory tract samples (in this context, nasal swabs were the most unrewarding specimen for detecting colonization with MDR Gram-negative bacteria) specially MDR *Enterobacterales*, surveillance of pharyngeal and nasal sites enabled us to document MDRB colonization, mostly by non-fermenting Gram-negative rods, that went undetected in axillary-rectal cultures in 25.4% of admissions.

Sex (male), underlying hematological malignancy (acute myeloid leukemia) and transplantation were associated with increased risk of MDRB colonization in univariate analysis, while a trend towards significance was observed in classically associated factors such as age and antibiotics treatment within the month prior to admission [1–3]. The impact of sex and acute myeloid leukemia on increasing the risk of colonization by MDRB has been previously reported in particular for carbapenemase-producing *Enterobacterales* [10, 14, 32–35].

In our view, the key observation of the current study was that overall, the MDRB-based BSI rate was significantly higher in MDRB-colonized patients than those who were not (*p* = 0.04). Moreover, among the 16 MDRB BSI episodes developing in MDRB-colonized patients, 13 were deemed to be caused by the colonizing isolate, principally ESBL-producing *Enterobacterales* and MDR *P. aeruginosa*. Indeed, MDRB colonization was independently associated with increased risk of MDRB BSI. Of these 13 isolates, 12 could be recovered from axillary-rectal cultures, while one (MDR-*P. aeruginosa*) was cultured from a pharyngeal specimen, but missed in axillary-rectal screening cultures. MDRB surveillance cultures, notably those from axillary-rectal sites, therefore offered valuable information to predict the antimicrobial susceptibility profile of BSI-causing MDRB in 81% of episodes. Our data concur to some extent with findings of several studies. Cattaneo and colleagues [12] conducted a multicenter prospective observational study involving 18 hematologic centers during a 6-month period; overall, 37 MDRB-colonized patients (25.7%) developed at least one BSI, of which 23 (16% of the whole series) developed BSI from the same MDRB pathogen with a rate of 15.6% for ESBL-producing *Enterobacterales* and 14.1% for carbapenemase-producing Gram-negative bacteria. The study concluded that empiric antibiotic treatment selection should be guided by known colonization in hematologic patients. Likewise, data from a study by Jaiswal et al. [10] demonstrated that hematologic patients colonized with carbapenemase-producing *Enterobacterales* have the highest risk of MDRB-BSI and mortality, particularly those with acute myeloid leukemia. In turn, Ballo et al*.* [11] highlighted the importance of exhaustive MDRB screening in acute myeloid patients with febrile neutropenia, as they require special surveillance due to their high risk of invasive infection and mortality. Liss et al*.* [13] found that colonization with ESBL-producing *Enterobacterales* was associated with increased risk ratios towards BSI development due to ESBL-producing *Enterobacterales*. Finally, Sadowska-Klasa et al. [17] retrospectively analyzed the data of 120 patients who underwent HCT for hematologic disorders and found that prior colonization was significantly correlated with MDRB infections (*p* < 0.001), especially bacteremia (*p* = 0.038).

The main limitation of the study is the relatively low number of registered MDRB BSI episodes. Nonetheless, the multi-body-site surveillance approach and MDRB pre-enrichment using selective broths, both of which may increase sensitivity for MDRB detection, can be considered strengths.

## Conclusion

In summary, our data indicated that MDRB colonization in a highly heterogeneous cohort of hematological patients is a frequent event associated with increased risk of MDRB BSI. Thus, MDRB colonization screening cultures may be useful for predicting MDRB BSI and tailoring empirical antibiotic treatments on an individual basis. The data also suggested that upper respiratory tract sampling for surveillance cultures adds little to axillary-rectal swabbing only in terms of predicting MDR Gram-negative BSI development.

## Data Availability

The datasets generated during and/or analyzed during the current study are available from the corresponding author on reasonable request.

## Abbreviations

AMR: Antimicrobial resistance
BHI: Brain–heart infusion broth
BSI: Bloodstream infections
ESBL: Extended-spectrum beta-lactamase
EUCAST: European Committee on Antimicrobial Susceptibility Testing
HR: Hazard ratio
ICU: Intensive care unit
MALDI-ToF–MS: Matrix-assisted laser desorption/ionization time-of-flight mass spectroscopy
MDRB: Multi-drug resistant bacteria
MIC: Minimum inhibitory concentration
MRSA: Methicillin-resistant *Staphylococcus aureus*
OR: Odss ratio
VIM: Verona integron-encoded metallo-beta-lactamase
VRE: Vancomycin-resistant enterococci

## References

[1] Gudiol C, Bodro M, Simonetti A (2013). Changing aetiology, clinical features, antimicrobial resistance, and outcomes of bloodstream infection in neutropenic cancer patients. Clin Microbiol Infect.

[2] Satlin MJ, Jenkins SG, Walsh TJ (2014). The global challenge of carbapenem-resistant Enterobacteriaceae in transplant recipients and patients with hematologic malignancies. Clin Infect Dis.

[3] Gudiol C, Tubau F, Calatayud L, Garcia-Vidal C, Cisnal M, Sanchez-Ortega I (2011). Bacteraemia due to multidrug-resistant Gram-negative bacilli in cancer patients: risk factors, antibiotic therapy and outcomes. J Antimicrob Chemother.

[4] Tumbarello M, Viale P, Viscoli C, Trecarichi EM, Tumietto F, Marchese A, Spanu T, Ambretti S, Ginocchio F, Cristini F, Losito AR, Tedeschi S, Cauda R, Bassetti M (2012). Predictors of mortality in bloodstream infections caused by *Klebsiella pneumoniae* carbapenemase-producing *K. pneumoniae*: importance of combination therapy. Clin Infect Dis.

[5] Trecarichi EM, Tumbarello M (2014). Antimicrobial-resistant Gram-negative bacteria in febrile neutropenic patients with cancer: current epidemiology and clinical impact. Curr Opin Infect Dis.

[6] Girmenia C, Rossolini GM, Piciocchi A (2015). Infections by carbapenem-resistant Klebsiella pneumoniae in SCT recipients: a nationwide retrospective survey from Italy. Bone Marrow Transplant.

[7] Lalaoui R, Javelle E, Bakour S, Ubeda C, Rolain JM (2020). Infections due to carbapenem-resistant bacteria in patients with hematologic malignancies. Front Microbiol.

[8] Weinstock DM, Conlon M, Iovino C, Aubrey T, Gudiol C, Riedel E, Young JW, Kiehn TE, Zuccotti G (2007). Colonization, bloodstream infection, and mortality caused by vancomycin-resistant enterococcus early after allogeneic hematopoietic stem cell transplant. Biol Blood Marrow Transplant.

[9] Pagano L, Caira M, Trecarichi EM, Spanu T, Di Blasi R, Sica S, Sanguinetti M, Tumbarello M (2014). Carbapenemase-producing *Klebsiella pneumoniae* and hematologic malignancies. Emerg Infect Dis.

[10] Jaiswal SR, Gupta S, Kumar RS, Sherawat A, Rajoreya A, Dash SK (2018). Gut colonization with carbapenem-resistant Enterobacteriaceae adversely impacts the outcome in patients with hematological malignancies: results of a prospective surveillance study. Mediterr J Hematol Infect Dis..

[11] Ballo O, Tarazzit I, Stratmann J, Reinheimer C, Hogardt M, Wichelhaus TA, Kempf V, Serve H, Finkelmeier F, Brandts C (2019). Colonization with multidrug resistant organisms determines the clinical course of patients with acute myeloid leukemia undergoing intensive induction chemotherapy. PLoS ONE.

[12] Cattaneo C, Di Blasi R, Skert C, Candoni A, Martino B, Di Renzo N, Delia M, Ballanti S, Marchesi F, Mancini V, Orciuolo E, Cesaro S, Prezioso L, Fanci R, Nadali G, Chierichini A, Facchini L, Picardi M, Malagola M, Orlando V, Trecarichi EM, Tumbarello M, Aversa F, Rossi G, Pagano L (2018). Bloodstream infections in haematological cancer patients colonized by multidrug-resistant bacteria. Ann Hematol.

[13] Liss BJ, Vehreschild JJ, Cornely OA, Hallek M, Fatkenheuer G, Wisplinghoff H (2012). Intestinal colonisation and blood stream infections due to vancomycin-resistant enterococci (VRE) and extended-spectrum beta-lactamase-producing Enterobacteriaceae (ESBLE) in patients with haematological and oncological malignancies. Infection.

[14] Kömürcü B, Tükenmez Tigen E, Toptaş T, Fıratlı Tuğlular T, Koten V (2020). Korten V Rectal colonization with multidrug-resistant gram-negative bacteria in patients with hematological malignancies: a prospective study. Expert Rev Hematol.

[15] Vehreschild MJ, Hamprecht A, Peterson L, Schubert S, Häntschel M, Peter S, Schafhausen P, Rohde H, Lilienfeld-Toal MV, Bekeredjian-Ding I, Libam J, Hellmich M, Vehreschild JJ, Cornely OA, Seifert H (2014). A multicentre cohort study on colonization and infection with ESBL-producing Enterobacteriaceae in high-risk patients with haematological malignancies. J Antimicrob Chemother.

[16] Alrstom A, Alsuliman T, Daher N, Abouharb R (2021). The impact of modifying empirical antibiotic therapy based on intestinal colonization status on clinical outcomes of febrile neutropenic patients. Infect Chemother.

[17] Sadowska-Klasa A, Piekarska A, Prejzner W, Bieniaszewska M, Hellmann A (2018). Colonization with multidrug-resistant bacteria increases the risk of complications and a fatal outcome after allogeneic hematopoietic cell transplantation. Ann Hematol.

[18] Tacconelli E, Cataldo MA, Dancer SJ, De Angelis G, Falcone M, Frank U, Kahlmeter G, Pan A, Petrosillo N, Rodríguez-Baño J, Singh N, Venditti M, Yokoe DS, Cookson B (2014). ESCMID guidelines for the management of the infection control measures to reduce transmission of multidrug-resistant Gramnegative bacteria in hospitalized patients. Clin Microbiol Infect.

[19] ECDC. European centre for disease prevention and control. Systematic review of the effectiveness of infection control measures to prevent the transmission of carbapenemase-producing Enterobacteriaceae through cross-border transfer of patients. 2014.

[20] Stockholm: ECDC.

[21] Royal College of Physicians of Ireland. Guidelines for the prevention and control of multi-drug resistant organisms (MDRO) excluding mrsa in the healthcare setting. 2012.

[22] Public Health England (2013) Acute trust toolkit for the early detection, management and control of carbapenemase-producing Enterobacteriaceae.

[23] Health Protection Scotland. Carbapenemase producing Enterobacteriaceae (CPE) prevention and management toolkit for inpatient areas. 2013.

[24] Magiorakos AP, Burns K, Rodríguez Baño J, Borg M, Daikos G, Dumpis U, Lucet JC, Moro ML, Tacconelli E, Simonsen GS, Szilágyi E, Voss A, Weber JT (2017). Infection prevention and control measures and tools for the prevention of entry of carbapenem-resistant Enterobacteriaceae into healthcare settings: guidance from the European Centre for Disease Prevention and Control. Antimicrob Resist Infect Control.

[25] Otter JA, Mutters NT, Tacconelli E, Gikas A, Holmes AH (2015). Controversies in guidelines for the control of multidrug-resistant Gram-negative bacteria in EU countries. Clin Microbiol Infect.

[26] Tormo N, Albert E, Borrajo E, Bosque M, Camarena JJ, Domínguez V, Fuentes E, Gascón I, Gomila B, Gonzalo N, Jiménez M, Martínez O, Nogueira JM, Orta N, Prat J, Rodríguez JC, Gimeno C, Navarro D (2018). A survey on practices for active surveillance of carriage of multidrug-resistant bacteria in hospitals in the Autonomous Community of Valencia, Spain. Eur J Clin Microbiol Infect Dis.

[27] Sánchez Carrillo C, Guerrero Gómez C, Cercenado E, Cantón R. Recogida, transporte y procesamiento general de muestras de laboratorio de Microbiología. Procedimientos de microbiología clínica. Recomendaciones de la Sociedad Española de Enfermedades Infecciosas y Microbiología Clínica (SEIMC). 2003.

[28] Torres I, Gimenez E, Pascual T, Bueno F, Huntley D, Martínez M, Navarro D (2017). Short-term incubation of positive blood cultures in brain-heart infusion broth accelerates identification of bacteria by matrix-assisted laser desorption/ionization time-of-flight mass-spectrometry. J Med Microbiol.

[29] Torres I, Palop N, Salvador RB, Gómez JB, Gimeno C, Navarro D (2019). Evaluation of the DNA microarray “AMR Direct Flow Chip Kit” for detection of antimicrobial resistance genes from Gram-positive and Gram-negative bacterial isolated colonies. Enferm Infecc Microbiol Clin.

[30] Magiorakos AP, Srinivasan A, Carey RB, Carmeli Y, Falagas ME, Giske CG, Harbarth S, Hindler JF, Kahlmeter G, Olsson-Liljequist B, Paterson DL, Rice LB, Stelling J, Struelens MJ, Vatopoulos A, Weber JT, Monnet DL (2012). Multidrug-resistant, extensively drug-resistant and pandrug-resistant bacteria: an international expert proposal for interim standard definitions for acquired resistance. Clin Microbiol Infect.

[31] Papadomichelakis E, Kontopidou F, Antoniadou A, Poulakou G, Koratzanis E, Kopterides P, Mavrou I, Armaganidis A, Giamarellou H (2008). Screening for resistant gram-negative microorganisms to guide empiric therapy of subsequent infection. Intensive Care Med.

[32] Ayats J, Corbella X, Ardanuy C, Domínguez MA, Ricart A, Ariza J, Martin R, Liñares J (1997). Epidemiological significance of cutaneous, pharyngeal, and digestive tract colonization by multiresistant *Acinetobacter baumannii* in ICU patients. J Hosp Infect.

[33] Micozzi A, Gentile G, Minotti C, Cartoni C, Capria S, Ballarò D, Santilli S, Pacetti E, Grammatico S, Bucaneve G, Foà R (2017). Carbapenem-resistant Klebsiella pneumoniae in high-risk haematological patients: factors favouring spread, risk factors and outcome of carbapenem-resistant *Klebsiella pneumoniae* bacteremias. BMC Infect Dis.

[34] Trecarichi EM, Pagano L, Candoni A, Pastore D, Cattaneo C, Fanci R, Nosari A, Caira M, Spadea A, Busca A, Vianelli N, Tumbarello M (2015). Current epidemiology and antimicrobial resistance data for bacterial bloodstream infections in patients with hematologic malignancies: an Italian multicentre prospective survey. Clin Microbiol Infect.

[35] Trecarichi EM, Pagano L, Martino B, Candoni A, Di Blasi R, Nadali G, Fianchi L, Delia M, Sica S, Perriello V, Busca A, Aversa F, Fanci R, Melillo L, Lessi F, Del Principe MI, Cattaneo C, Tumbarello M (2016). Bloodstream infections caused by *Klebsiella pneumoniae* in onco-hematological patients: clinical impact of carbapenem resistance in a multicentre prospective survey. Am J Hematol.

